# Patterns and prognostic predictive value of perineural invasion in esophageal squamous cell carcinoma

**DOI:** 10.1186/s12885-022-10386-w

**Published:** 2022-12-08

**Authors:** Yu Ma, Jie Chen, Xi Yao, Zhenzhen Li, Wensheng Li, Hongtao Wang, Jianfei Zhu

**Affiliations:** 1 Department of Pathology, Shaanxi Provincial People’s Hospital, Xi’an, 710068 People’s Republic of China; 2 Department of Anesthesiology, Shaanxi Provincial People’s Hospital, Xi’an, 710068 People’s Republic of China; 3 Department of Thoracic Surgery, Shaanxi Provincial People’s Hospital, Xi’an, 710068 People’s Republic of China

**Keywords:** Perineural invasion, Esophageal squamous cell carcinoma, TNM stage, Prognosis

## Abstract

**Background:**

The pathological phenotype of perineural invasion (PNI) in squamous cell carcinoma (ESCC) is prevalent but highly heterogeneous.

**Methods:**

Postoperative specimens from all patients with ESCC at Shaanxi Provincial People’s Hospital were evaluated for PNI using haematoxylin and eosin (H&E) staining and S100 immunohistochemistry (IHC). We determined the correlation between PNI status and clinical outcomes.

**Results:**

Among 349 ESCC cases, PNI was identified in 127 patients (36.3%), and four subtypes of PNI were identified in our study. Correlation analysis confirmed that PNI was related to tumour invasion depth (pT stage) and lymph node status (pN stage) (*P* < 0.05). Multivariate analysis showed that PNI (*P* = 0.001) was an independent factor affecting disease-free survival (DFS) in ESCC, and a similar result was found for overall survival (OS) (*P* = 0.017). Further analysis revealed that PNI status was a prognostic factor of DFS (*P* < 0.001) and OS (*P* = 0.003) exclusively in pN-negative patients. We also found that patients with the PNI-a subtype had better DFS (*P* = 0.002) and OS (*P* = 0.002) than patients with the other three subtypes (PNI-b, c, d).

**Conclusion:**

The pathological phenotypes of PNI are diverse, and the identification of PNI subtypes has important clinical guiding value.

## Introduction

Esophageal cancer is the sixth leading cause of death due to malignant tumours with aggressive biological behaviour [1]. Its pathological subtypes have obvious geographical distribution characteristics. Esophageal squamous cell carcinoma (ESCC) is the main type observed in China and East Asia, whereas esophageal adenocarcinoma (EA) is the main type noted in Europe and America [2–5]. Despite continuous updates of the TNM classification system and the application of multidisciplinary treatment (MDT) in recent years, the prognosis of patients remains poor, with a total 5-year overall survival (OS) rate of 30.0% [6, 7]. Previous studies have confirmed that tumour microinvasion is an important early warning event for metastasis and recurrence of operable ESCC [8, 9]. Microinvasion of ESCC, including vascular invasion (VI) and lymphatic invasion (LI), could predict the prognosis of ESCC and exacerbate its TNM staging [10, 11]. Therefore, early identification of its invasion pattern is important for ESCC diagnosis and treatment. Some studies have confirmed that perineural invasion (PNI) also plays an important role in ESCC invasion and metastasis, and the pathological phenotypes of PNI are diverse [12, 13]. At present, there is a lack of relevant research on the pathological subtypes of PNI in ESCC. Therefore, this study aimed to explore the relationship between PNI pathological subtypes and clinical outcomes of patients with ESCC.

## Materials and methods

### Research design

**Fig. 1:**
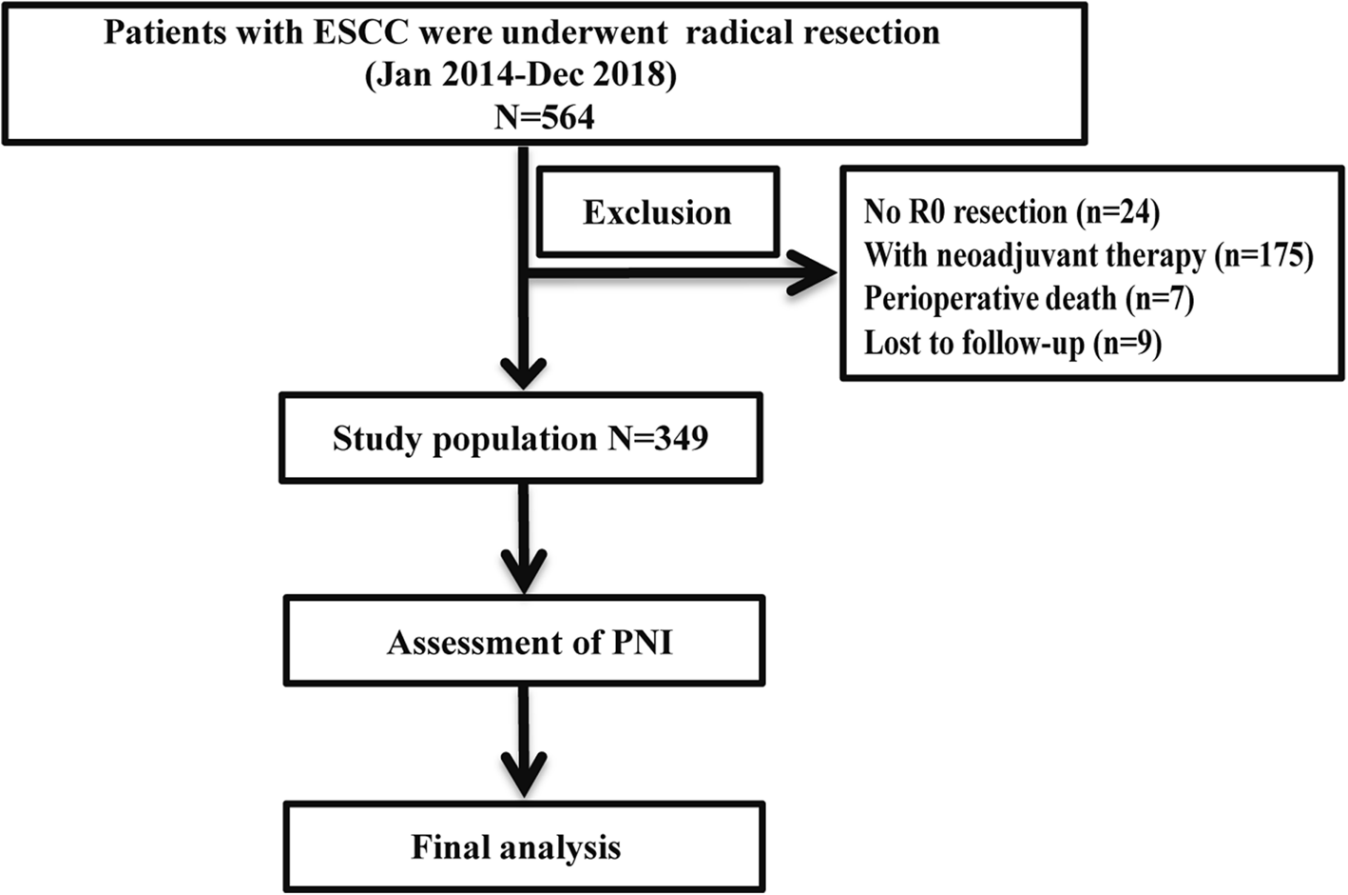
A flowchart of this study

A retrospective analysis of patients who underwent radical oesophagectomy between January 2014 and December 2018 was conducted (Fig. 1). The inclusion criteria were as follows: (1) pathological diagnosis of ESCC; (2) no antitumour treatment before surgery; (3) R0 resection; (4) no history of malignant tumour; and (5) all postoperative specimens underwent PNI assessment. The patients were followed up regularly in accordance with the NCCN guidelines, and the last follow-up was performed in October 2021.

### Assessment of PNI

**Fig. 2:**
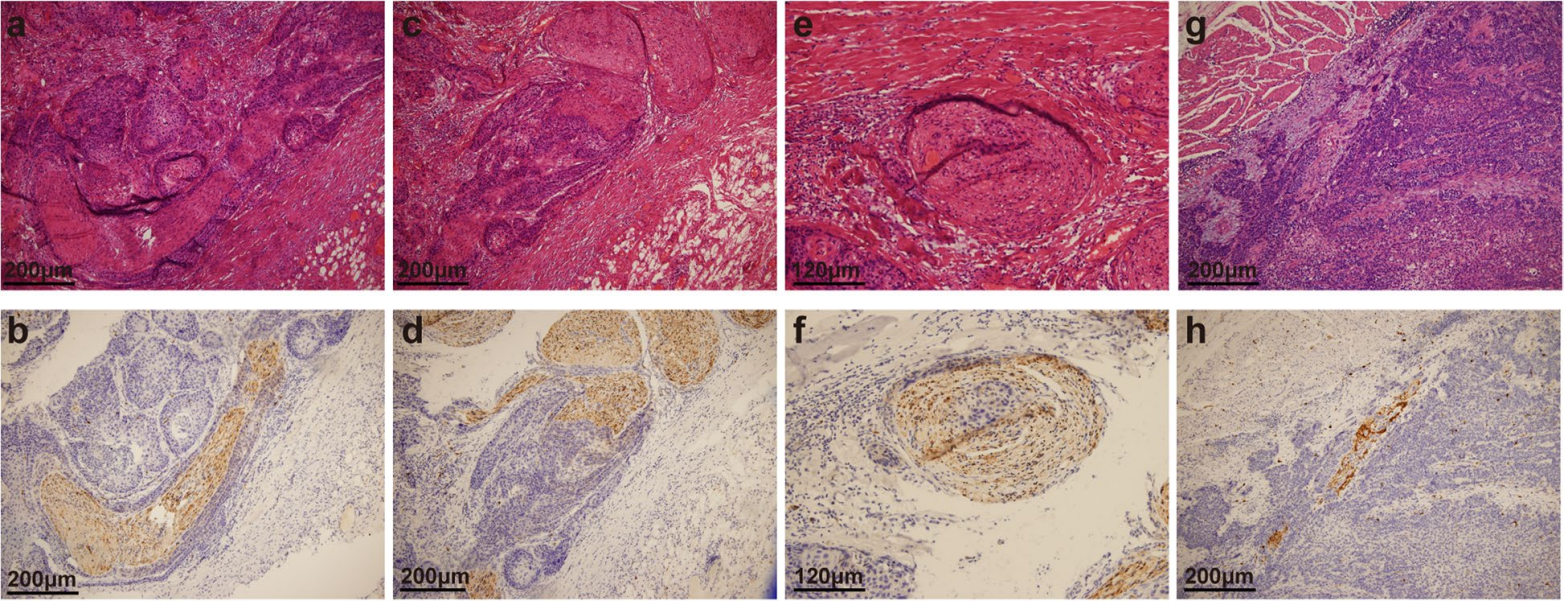
Representative histological characteristics of PNI in ESCC. Typical features of HE staining (**a**) and IHC staining (**b**) of PNI-a; typical features of HE staining (**c**) and IHC staining (**d**) of PNI-b; typical features of HE staining (**e**) and IHC staining (**f**) of PNI-c; typical features of HE staining (**g**) and IHC staining (**h**) of PNI-d. PNI, perineural invasion; ESCC, oesophageal squamous cell carcinoma; H&E, haematoxylin and eosin; IHC, immunohistochemistry

Haematoxylin and eosin (H&E) staining and immunohistochemistry (IHC) of PNI sections were reviewed without the knowledge of the pathology report and re-evaluated for PNI by independent attending pathologists (Yu Ma, Jie Chen, Zhenzhen Li, and Wensheng Li) blinded to the patients’ outcomes. IHC was performed using S-100 (ready-to-use, China Maixin) monoclonal antibody to evaluate nerves using an automatic immunostainer and Ultra VISION universal DAB (3,3′-diaminobenzidine) detection kit (Ventana Medical Systems, Inc. Tucson, AZ, USA). The nerve fibre could be clearly identified in the serial section by positive S100 IHC staining. Appendix tissue sections were used as the positive and negative controls. All microscopic analyses were performed using an optical microscope (Zeiss, Germany). The diagnostic criteria [14, 15] for PNI were as follows: (1) when cancer cells are not located in the nerve sheath but close to the nerve in the perineural environment, greater than one-third of the nerve circumference should be surrounded by cancer cells; (2) cancer cells should be immersed in the nerve sheath. Furthermore, we divided PNI into four subtypes and defined these subtypes based on the pathological characteristics of PNI. PNI-a is defined as cancer cells tightly surrounding nerve fibres, and the perineurium exhibits high integrity. PNI-b is defined as cancer cells invading nerve fibres and destroying the perineurium. PNI-c is defined as cancer cells invading the centre of nerve fibres. PNI-d is defined as cancer cells irregularly destroying nerve fibres and breaking through the perineurium (Fig. 2).

### Statistical analysis

The relationship between PNI and clinicopathological characteristics was tested using the chi-square test. The Kaplan–Meier method was used to calculate disease-free survival (DFS) and overall survival (OS), and the difference in survival rates between the groups was tested using the log-rank test. All statistically significant prognostic factors identified in the univariate analysis were included in the multivariate Cox regression analysis, and stepwise regression and collinearity tests were used in our analysis. The chi-square test was used to assess the fit of the Cox proportional hazards model. A two-tailed test with a *P* value of < 0.05 was considered statistically significant. All statistical analyses were performed using SPSS 22.0 and Stata 15.0.

## Results

### Patient characteristics

Overall, 349 patients with ESCC, including 255 men and 94 women, were included in this study. Regarding tumour location, patients with mid-thoracic esophageal cancer were the most prevalent (223/349, 63.9%). All patients were updated according to the eighth edition of the TNM staging criteria. In total, 44, 145, and 147 patients were diagnosed with stage I, II, and III disease, respectively. Notably, 15 patients were upgraded from stage III to stage IV by the new staging system and were also included in the analysis (Table 1).

**Table 1:** Correlation between perineural invasion and clinicopathological characteristics of esophageal squamous cell carcinoma

Variable	Total	Perineural invasion		*P* value
	349	Present(%)	Absent(%)	
Sex				0.432
Man	255	94(36.9)	161(63.1)	
Woman	94	33(35.1)	61(64.9)	
Age				0.496
<65 years	287	105(36.6)	182(63.4)	
≥ 65 years	62	22(35.5)	40(64.5)	
Tumor location				
Upper	29	7(24.1)	22(75.9)	0.195
Middle	223	88(39.5)	135(60.5)	
Lower	97	32(33.0)	65(67.0)	
Tumor length				0.314
≤ 4.0 cm	175	61(34.9)	114(65.1)	
>4.0 cm	174	66(37.9)	108(62.1)	
Differentiation				0.150
G1	106	46(43.4)	60(56.6)	
G2	161	51(31.7)	110(68.3)	
G3	82	30(36.6)	52(63.4)	
p T stage				
T1	38	7(18.4)	31(81.6)	0.010
T2	70	19(27.1)	51(72.9)	
T3	233	97(41.6)	136(58.4)	
T4	8	4(50.0)	4(50.0)	
p N stage				<0.001
N0	189	50(26.5)	139(73.5)	
N1	90	40(44.4)	50(55.6)	
N2	58	31(53.4)	27(46.6)	
N3	12	6(50.0)	6(50.0)	
p TNM stage				<0.001
I stage	44	8(18.2)	36(81.8)	
II stage	145	42(29.0)	103(71.0)	
III stage	147	70(47.6)	77(52.4)	
IV stage	13	7(53.8)	6(46.2)	

### PNI was related to pathological stage features of ESCC

The overall prevalence of PNI among ESCC cases in our study was 36.3% (127/349). Four types of PNI (PNI-a, PNI-b, PNI-c, and PNI-d) were identified: 35 cases of PNI-a, 32 cases of PNI-b, 36 cases of PNI-c, and 24 cases of PNI-d. Next, we analysed the relationship between PNI and the clinicopathological characteristics of patients with ESCC; the details are listed in Table 1. As the depth of tumour invasion increased (pT stage), the occurrence of PNI gradually increased (*P* = 0.010). We also found that PNI was associated with lymph node metastasis (pN stage). The incidence of PNI in patients with positive lymph nodes was as high as 48.1% compared with 26.5% in patients with negative lymph nodes (*P* < 0.001).

### Prognostic value of PNI in ESCC

**Fig. 3:**
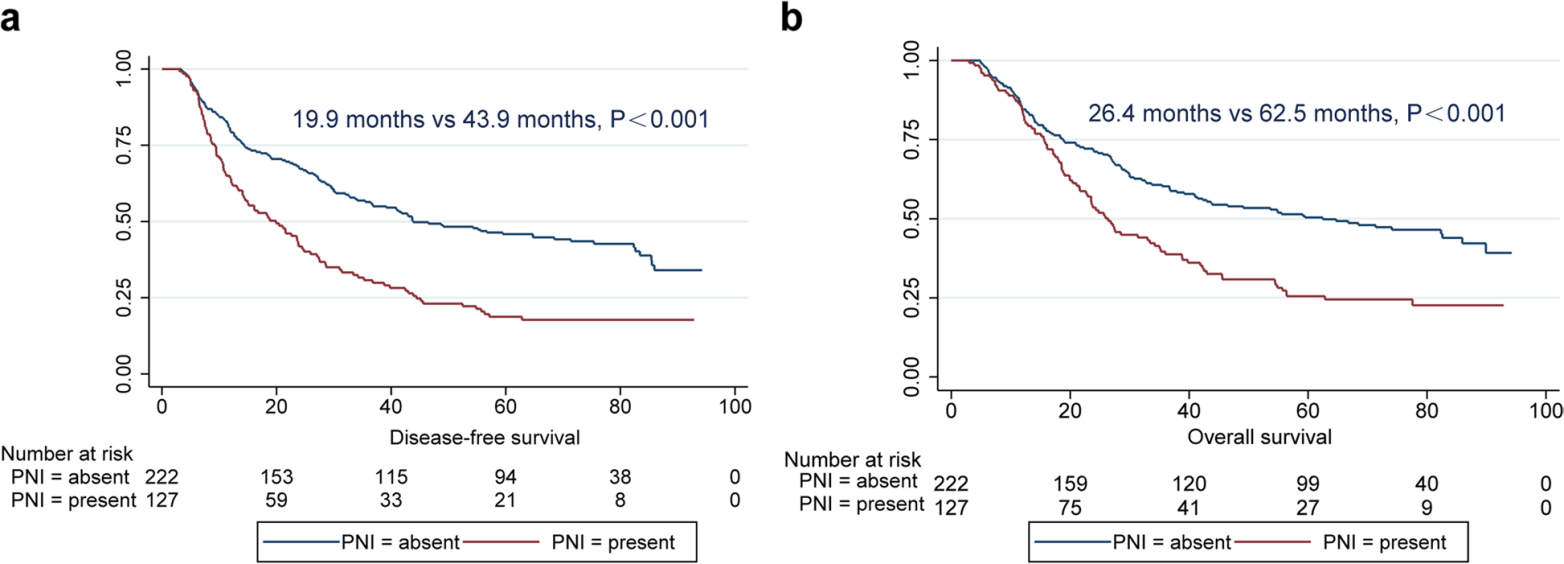
Survival curves of ESCC patients with different PNI statuses. **a** DFS of ESCC according to PNI status. **b** OS of ESCC according to PNI status. ESCC, oesophageal squamous cell carcinoma; PNI, perineural invasion; DFS, disease-free survival; OS, overall survival

Survival analysis showed that the median DFS time of patients with ESCC with PNI was significantly lower than that of patients without PNI (*P* < 0.001; Table 2 and Fig. 3a). Univariate analysis also showed that sex (*P* = 0.037), tumour length (*P* = 0.002), pT stage (*P* = 0.001), pN stage (*P* < 0.001), and pTNM stage (*P* < 0.001) were all important variables that affected the DFS of patients with ESCC. We included the five variables of sex, tumour length, pT stage, pN stage and PNI identified above in a multivariate Cox model for multivariate analysis (likelihood ratio: 2375, degree of freedom: 5, chi-square value: 60.8, overall *p* value≤0.001. Multivariate analysis showed that PNI (*P* = 0.001) and pN stage (*P* < 0.001) were independent factors affecting DFS in ESCC. Further analysis showed that in patients with ESCC, the presence of PNI increased the risk of recurrence and metastasis by 1.6-fold (hazard ratio [HR]: 1.6, 95% confidence interval [CI]: 1.2–2.1, *P* = 0.001) (Table 3).

**Table 2:** Univariate analysis of DFS and OS in esophageal squamous cell carcinoma

Variable	DFS			OS		
	Median	95%CI	*P*	Median	95%CI	*P*
Sex			0.037			0.027
Man	28.6	21.8–35.5		35.2	27.2–43.3	
Woman	42.9	22.3–63.4		55.7	21.9–89.6	
Age			0.426			0.329
<65 years	33.4	25.1–41.7		42.3	30.5–54.0	
≥ 65 years	26.4	20.5–32.4		30.0	20.4–39.4	
Tumor location			0.699			0.670
Upper	27.2	21.0–33.3		35.2	19.3–51.2	
Middle	35.3	26.7–43.9		42.5	30.1–5.9	
Lower	27.2	19.5–35.0		31.4	10.5–52.2	
Tumor length			0.002			0.013
≤ 4.0 cm	42.9	32.4–53.3		49.1	36.9–61.4	
>4.0 cm	26.4	20.7–32.2		29.9	19.4–40.4	
Differentiation			0.799			0.664
G1	30.0	19.8–40.2		35.0	23.1–47.0	
G2	32.0	21.2–42.9		41.9	25.8–57.9	
G3	35.3	20.0–50.5		45.6	25.8–65.4	
p T stage			0.001			
T1	85.9	46.2–125.7		85.9	NA	0.002
T2	41.4	33.0–49.8		43.9	11.1–76.6	
T3	26.8	20.9–32.8		32.6	25.2–40.0	
T4	14.3	0.0–80.3		20.0	0.0–79.9	
p N stage			<0.001			<0.001
N0	59.5	34.4–84.6		82.6	58.6–106.6	
N1	25.9	18.2–33.7		36.7	24.9–48.5	
N2	13.5	10.3–16.7		17.9	12.8–23.0	
N3	8.8	2.0–15.5		11.8	9.8–13.7	
p TNM stage			<0.001			<0.001
I stage	71.4	43.5–99.3		85.9	NA	
II stage	55.7	26.3–85.1		66.6	57.4–97.8	
III stage	18.8	11.7–25.9		45.7	21.5–31.3	
IV stage	10.8	4.9–16.6		11.9	9.9–14.0	
Perineural invasion			<0.001			<0.001
Present	19.9	13.1–26.7		26.4	22.1–30.7	
Absent	43.9	26.4–61.3		62.5	39.0–85.9

**Table 3:** Multivariate analysis of DFS and OS in esophageal squamous cell carcinoma

Variable	DFS			OS		
	RR	95%CI	*P*	RR	95%CI	*P*
Sex: Woman/Man	0.8	0.6–1.1	0.150	0.8	0.6–1.1	0.132
Tumor length: >4.0 cm/≤4.0 cm	1.2	0.9–1.5	0.266	1.1	0.8–1.4	0.713
Perineural invasion: Present/ Absent	1.6	1.2–2.1	0.001	1.4	1.1–1.9	0.017
p T stage: T3 + T4/T1 + T2	1.3	0.9–1.7	0.136	1.3	1.0–1.9	0.083
p N stage: N0/N1 + N2 + N3	2.0	1.5–2.6	<0.001	2.0	1.5–2.7	<0.001

Similarly, patients with ESCC harbouring PNI had poorer OS than those without PNI (26.4 months vs. 62.5 months, *P* < 0.001; Fig. 3b). We also found that sex (*P* = 0.027), tumour length (*P* = 0.013), pT stage (*P* = 0.002), pN stage (*P* < 0.001), and pTNM stage (*P* < 0.001) can serve as predictors of OS in ESCC (Table 2). The Cox model consists of five variables. The likelihood ratio for this model was 2172 with 5 degrees of freedom. The chi-square value was 53.0, and the overall *p* value≤0.001. Multivariate analysis showed that PNI (HR: 1.4, 95% CI: 1.1–1.9, *P* = 0.017) and pN (HR: 2.0, 95% CI: 1.5–2.7, *P* < 0.001) were independent factors predictive of OS in ESCC (Table 3).

**Fig. 4:**
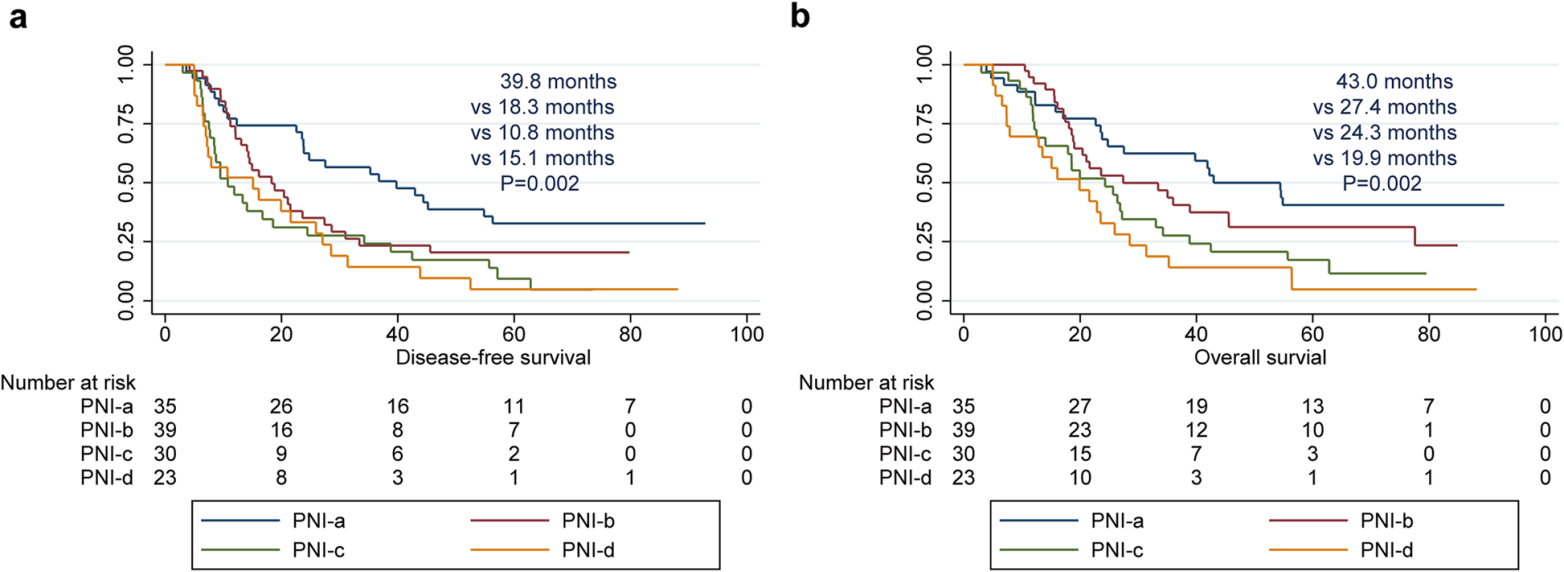
Survival curves of ESCC patients with different PNI subtypes. **a** DFS of ESCC according to different PNI subtypes; **b** OS of ESCC according to different PNI subtypes. ESCC, oesophageal squamous cell carcinoma; PNI, perineural invasion; DFS, disease-free survival; OS, overall survival

Moreover, we observed a correlation between PNI phenotypes and clinicopathological characteristics (Table 4). We also analysed the impact of PNI subtypes on the prognosis of patients with ESCC (Fig. 4). Among the four subtypes, patients with the PNI-a subtype had better DFS (*P* = 0.002) and OS (*P* = 0.002) than patients with the other three subtypes (PNI-b, c, d).

**Table 4:** The correlation between PNI phenotypes and clinicopathological characteristics of esophageal squamous cell carcinoma

Variable	Total	Subtypes of PNI				*P* value
	127	PNI-a	PNI-b	PNI-c	PNI-d	
Sex						0.285
Man	94	26(27.7%)	29(30.9%)	19(20.2%)	20(21.2%)	
Woman	33	9(27.3%)	10(30.3%)	11(33.3%)	3(9.1%)	
Age						0.515
<65 years	105	27(25.7%)	33(31.4%)	27(25.7%)	18(17.2%)	
≥ 65 years	22	8(36.4%)	6(27.3%)	3(13.6%)	5(22.7%)	
Tumor location						0.007
Upper	7	1(14.3%)	0(0)	2(28.6%)	4(57.1%)	
Middle	88	29(33.0%)	30(34.1%)	20(22.7)	9(10.2%)	
Lower	32	5(15.6%)	9(28.1%)	8(25.0%)	10(31.3%)	
Tumor length						0.189
≤ 4.0 cm	61	14(23.0%)	21(34.4%)	18(29.5%)	8(13.1%)	
>4.0 cm	66	21(31.8%)	18(27.3%)	12(18.2%)	15(22.7%)	
Differentiation						0.584
G1	46	15(32.6%)	12(26.1%)	9(19.6%)	10(21.7%)	
G2	51	12(23.5%)	15(29.4%)	16(31.4%)	8(15.7%)	
G3	30	8(26.6%)	12(40.0%)	5(16.7%)	5(16.7%)	
p T stage						0.782
T1	7	1(14.3%)	3(42.8%)	1(14.3%)	2(28.6%)	
T2	19	8(42.1%)	5(26.3%)	3(15.8%)	3(15.8%)	
T3	97	24(24.7%)	30(30.9%)	25(25.8%)	18(18.6%)	
T4	4	2(50.0%)	1(25.0%)	1(25.0%)	0(0)	
p N stage						0.144
N0	50	13(26.0%)	20(40.0%)	8(16.0%)	9(18.0%)	
N1	40	14(35.0%)	10(25.0%)	9(22.5%)	7(17.5%)	
N2	31	8(25.8%)	9(29.0%)	9(29.0%)	5(16.2%)	
N3	6	0(0)	0(0)	4(66.7%)	2(33.3%)	
p TNM stage						0.151
I stage	8	1(12.5%)	5(62.5%)	1(12.5%)	1(12.5%)	
II stage	42	11(25.6%)	15(35.7%)	7(16.7%)	9(21.4%)	
III stage	70	23(32.9%)	18(25.7%)	18(25.7%)	11(15.7%)	
IV stage	7	0(0)	1(14.3%)	4(57.1%)	2(28.6%)

### PNI as a potential supplement classification for pN-negative ESCC patients

**Fig. 5:**
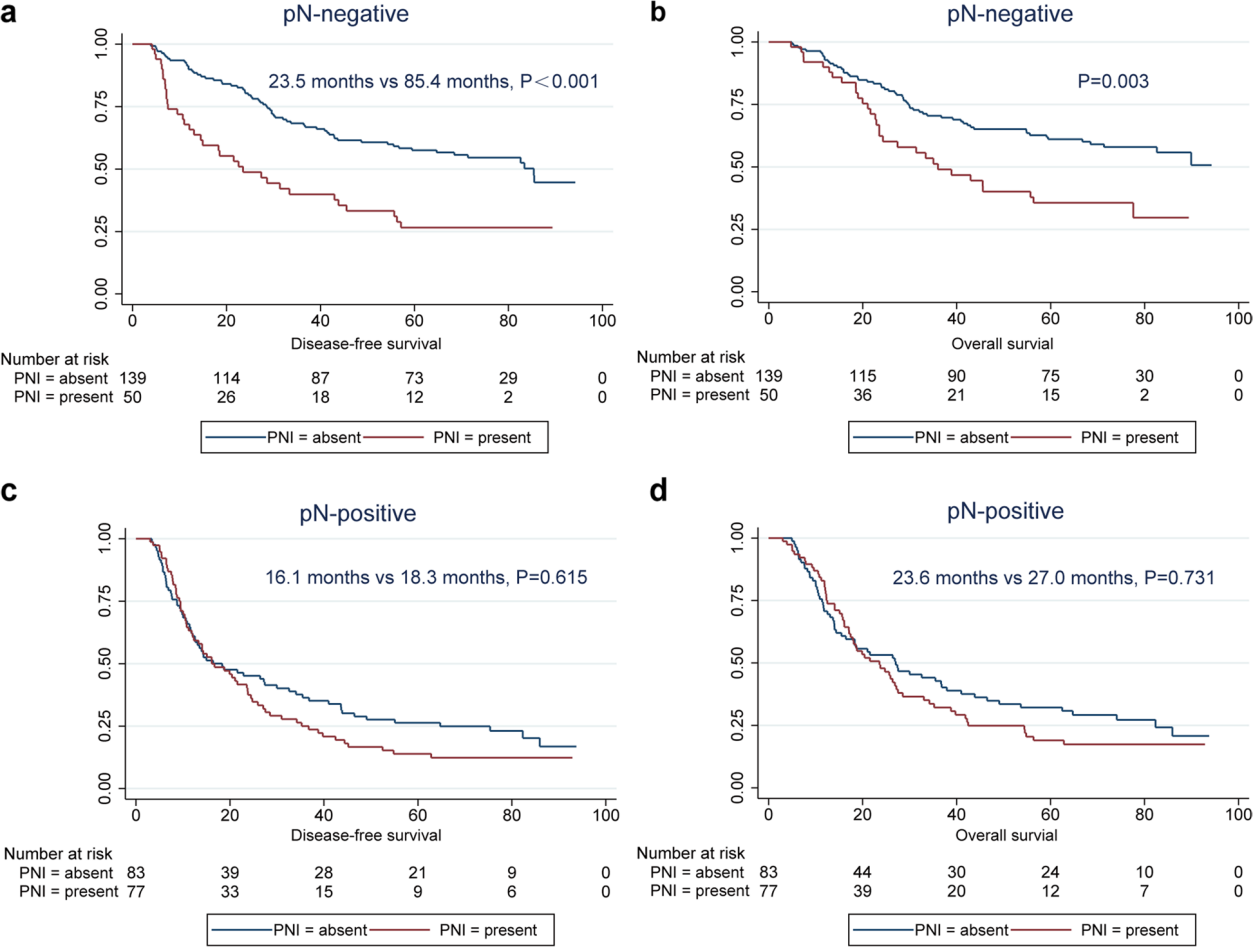
Effects of PNI status on ESCC survival in different pN stages. **a** DFS of patients with pN-negative ESCC according to PNI status; **b** OS of patients with pN-negative ESCC according to PNI status; **c** DFS of patients with pN-positive ESCC according to PNI status; **d** OS of patients with pN-positive ESCC according to PNI status. ESCC, oesophageal squamous cell carcinoma; PNI, perineural invasion; DFS, disease-free survival; OS, overall survival

Next, we analysed the predictive role of PNI in the prognosis of patients with different pN stages of ESCC. For pN-negative patients, PNI was an influencing prognostic factor for DFS (*P* < 0.001) and OS (*P* = 0.003). However, for pN-positive patients, different PNI statuses did not affect DFS (*P* = 0.615) or OS (*P* = 0.731) (Fig. 5). These findings suggest that PNI status should be evaluated in patients with pN0 stage ESCC.

## Discussion

The mode of microinvasion of malignant tumours mainly involves VI and LI. PNI is an under recognized but very important tumour invasion route. Research on the pathological characteristics and prognostic value of PNI has been reported in a variety of tumours, the most common being pancreatic cancer and prostate cancer [16–18]. Whether PNI can be used as a factor to predict ESCC prognosis remains controversial [12, 19]. In the current study, our findings suggest that PNI may serve as a potential prognostic predictor in ESCC.

The incidence of PNI in ESCC varies from 7.9 to 50.7% give the lack of standard diagnostic criteria [12, 20, 21]. One study by a Chinese scholar found that the incidence of PNI in ESCC was as high as 47.7%. A possible reason for this high value was that the author applied a broader definition of PNI, which included the following two conditions: at least one-third of the nerves surrounding cancer cells do not invade the nerve sheath and cancer cells are present in any of the three layers of the nerve sheath [12]^13^. Xu et al. [20] reported that the use of H&E staining combined with S100 staining could significantly improve the accuracy of PNI diagnosis. Consistent with a previous study, in our study, 36.3% of ESCC cases were positive for PNI based on H&E combined with S100 staining. The pathological types of PNI were diverse, and different pathological subtypes had different prognostic features, suggesting that more detailed PNI typing should be performed in ESCC.

The PNI status of ESCC should be evaluated in clinical practice. Although PNI was considered to be an independent prognostic factor of ESCC in some studies [22, 23], Ochiai et al. [24] reported that PNI was not significantly correlated with the OS of esophageal cancer. To address the above questions, Gao et al. [25] designed a meta-analysis that included a total of 2770 patients with esophageal cancer undergoing surgery in 13 cohorts. The results confirmed that PNI was a poor prognostic biomarker for esophageal cancer and esophagogastric junction carcinoma. In the present study, we concluded that PNI is related to the TNM staging of ESCC, and PNI may be used as a supplemental prognostic factor, especially for pN-negative patients.

This study had certain limitations, which mainly include the following three aspects. First, the assessment of PNI before and after neoadjuvant therapy was not conducted in this study. Second, single-centre research may lead to bias in the sample selection. Third, although we used H&E and anti-S100 IHC to evaluate PNI, there are currently no definite diagnostic criteria for PNI. These issues require further investigation.

## Conclusion

In summary, PNI exhibited a nonnegligible invasion pattern in early-stage ESCC. PNI may serve as a potential supplement to the pathological TNM staging of ESCC and may serve as a prognostic predictor of ESCC.

## Data Availability

The datasets used during the current study are available from the corresponding author on reasonable request.

## Abbreviations

PNI: Perineural invasion
ESCC: Esophageal squamous cell carcinoma
EA: Esophageal adenocarcinoma
H&E: Haematoxylin and eosin
IHC: Immunohistochemistry
DFS: Disease-free survival
OS: Overall survival
VI: Vascular invasion
LI: Lymphatic invasion
